# Retrospective Post-Hospitalisation COVID-19 Mortality Risk Assessment of Patients in South Africa

**DOI:** 10.3390/ejihpe13090120

**Published:** 2023-09-01

**Authors:** Alexander Boateng, Daniel Maposa, Reshoketswe Mokobane

**Affiliations:** 1 Department of Biostatistics, University of the Free State, Bloemfontein 9300, South Africa; boatenga@ufs.ac.za; 2 Department of Statistics and Operations Research, University of Limpopo, Polokwane 0727, South Africa; reshoketswe.mokobane@ul.ac.za

**Keywords:** survival model, COVID-19, parametric models, semi-parametric frailty model

## Abstract

***Background***: This study explores the determinants impacting the mortality risk of COVID-19 patients following hospitalisation within South Africa’s Limpopo province. ***Methods***: Utilising a dataset comprising 388 patients, the investigation employs a frailty regression model to evaluate the influence of diverse characteristics on mortality outcomes, contrasting its performance against other parametric models based on loglikelihood measures. ***Results***: The findings underscore diabetes and hypertension as notable contributors to heightened mortality rates, underscoring the urgency of effectively managing these comorbidities to optimise patient well-being. Additionally, regional discrepancies come to the fore, with the Capricorn district demonstrating elevated mortality risks, thereby accentuating the necessity for precisely targeted interventions. Medical interventions, particularly ventilation, emerge as pivotal factors in mitigating mortality risk. Gender-based distinctions in mortality patterns also underscore the need for bespoke patient care strategies. ***Conclusions***: Collectively, these outcomes supply practical insights with implications for healthcare interventions, policy formulation, and clinical strategies aimed at ameliorating COVID-19 mortality risk among individuals discharged from hospitals within South Africa’s Limpopo province.

## 1. Introduction

The severe acute respiratory syndrome coronavirus-2 (SARS-CoV-2) that causes the coronavirus disease-2019 (COVID-19) is a novel coronavirus that has a high fatality and morbidity rate [1]. After receiving reports of 118,000 cases and 4291 deaths in 114 nations, the World Health Organization (WHO) declared COVID-19 a pandemic on 11 March 2020 [2]. Globally, as of 15 July 2022, there were more than 6.4 million reported fatalities and over 565.6 million confirmed cases [1]. As the novel coronavirus continues to evolve, there are still many limitations to our knowledge of who exactly this virus would impact critically.

In light of the current circumstances surrounding the global COVID-19 pandemic, this study delves into the intricacies of post-hospitalisation mortality risk among patients in South Africa’s Limpopo province. The unprecedented challenges posed by the pandemic have necessitated a thorough understanding of the factors influencing patient outcomes. By investigating these factors, we aim to shed light on the complexities of COVID-19 mortality risk, offering insights that can aid healthcare professionals, policymakers, and researchers in navigating the evolving landscape of patient care.

Indeed, the COVID-19 pandemic has prompted extensive research into understanding the disease’s clinical manifestations, risk factors, and outcomes. Mortality risk assessment is a pivotal aspect of this research, aiming to identify the factors contributing to higher mortality rates among patients hospitalised due to laboratory-confirmed COVID-19. Theoretical frameworks have underscored several critical concepts in this context. For instance, clinical severity and the presence of comorbidities have been identified as key factors influencing outcomes. Severe cases that necessitate hospitalisation are often linked to adverse outcomes, while patients with pre-existing conditions such as diabetes, cardiovascular diseases, and respiratory ailments face an elevated mortality risk [3,4].

Additionally, the immune response to COVID-19 plays a pivotal role in disease progression and outcomes. Dysregulated immune responses, including cytokine storms, have been associated with severe cases and poor outcomes [5]. Furthermore, age and gender are established risk factors for severe outcomes. Older adults are more vulnerable, and gender differences in mortality rates have been observed, with males generally experiencing higher mortality [6].

A number of studies have investigated the factors that influence post-hospitalisation mortality risk. For instance, [7] examined a cohort of COVID-19 patients and found that advanced age, high Sequential Organ Failure Assessment (SOFA) scores, and elevated d-dimer levels were associated with increased mortality risk post-hospitalisation.

Another significant study by [8] identified age, male sex, and various comorbidities (such as diabetes, obesity, and cardiovascular diseases) as independent risk factors for COVID-19-related mortality. Another study by [9] highlighted ethnic and racial disparities that impact mortality risk, with Black and Asian individuals having higher mortality risks even after adjusting for age, sex, and comorbidities.

On 27 March 2020, South Africa experienced its first COVID-19 fatality [10]. Since then, the nation has recorded 88.9 thousand citizen deaths due to the pandemic. Furthermore, 839 people died on 19 January 2021, the largest daily death reported during the outbreak. South Africa was the most affected nation in the region as of 24 October 2021, with more than 2.91 million infection cases [11]. The South Africa National Institute for Communicable Diseases (NICD) verified the country’s first COVID-19 case on 5 March 2020 [10,11]. Since then, the South African government has successfully implemented a robust and successful national response to stop COVID-19 (see SAResponsetoCOVID-19, 2020, for main steps and dates [10]).

Be that as it may, it has become crucial to look back and examine the mortality and associated risk factors among COVID-19 hospitalised patients in the Limpopo province of South Africa as we continue to count the costs of the devastating effects of the pandemic. Indeed, pinpointing the causes of mortality among COVID-19 patients in both urban and rural contexts within the province could help us better understand the disease and the necessity of these findings in developing efficient preventive and therapeutic strategies for future pandemics.

Several studies have identified age, underlying medical conditions, severity of illness, and laboratory markers as significant risk factors associated with mortality rate [12,13]. However, there are still gaps in the literature regarding the long-term outcomes of COVID-19 patients, especially those who survive hospitalisation. Most studies have focused on short-term outcomes, such as mortality rates during hospitalisation, and there is a need for more research on the long-term outcomes of COVID-19 patients. Again, some studies have only included patients from specific regions or populations, limiting their findings’ generalisability. Generally, more research is needed to fully understand the risk factors associated with mortality among hospitalised COVID-19 patients and to improve the accuracy of predicting patient outcomes. The study aims to conduct a retrospective post-hospitalisation COVID-19 mortality risk assessment of patients in the Limpopo province of South Africa.

It is envisaged that the findings of this study will help improve our understanding of the factors that influence mortality rate among hospitalised COVID-19 patients and help us identify potential interventions that may improve patient outcomes. The outline of the paper is as follows: literature review and materials and methods are presented in Section 2 and Section 3, respectively. The results and discussions are presented in Section 4, ending with the concluding remarks in Section 5.

## 2. Literature Review

The COVID-19 pandemic has spurred extensive research aimed at understanding the clinical aspects, risk factors, and management strategies for patients affected by the virus. In this context, several studies have sought to uncover predictive factors for COVID-19 severity, mortality risk, and the specific implications for patients with comorbidities such as diabetes mellitus and hypertension.

Zhou et al. (2021) employed machine learning techniques to analyse longitudinal measurements and identified eleven routine clinical features that effectively predict the severity of COVID-19 cases, offering valuable insights into risk assessment [14]. Similarly, Onder et al. (2020) examined case-fatality rates in Italy, elucidating characteristics associated with patients who succumbed to COVID-19 and contributing to understanding disease outcomes [15].

The management of diabetes mellitus (DM) amid the COVID-19 pandemic presented unique challenges. Koliaki et al. (2020) discussed practical issues and concerns surrounding the clinical management of DM patients during the pandemic [16]. In their research, [17] focused on inpatient COVID-19 mortality risk assessments, specifically exploring the interplay of diabetes mellitus. Their study employed interpretable machine learning models, enhancing the understanding of mortality risks in patients with diabetes.

The intersection of COVID-19 and diabetes mellitus has been a topic of significant interest. Investigating mortality outcomes, [18] highlighted that patients with both COVID-19 and diabetes face increased mortality risks, emphasizing the importance of tailored interventions. Yang et al. (2020) developed predictive models for clinical deterioration among COVID-19 patients, utilizing machine learning and readily available clinical data to enhance prognosis accuracy [19].

In another dimension, short-term outcomes of newly diagnosed diabetes in COVID-19 patients were explored by Zhou et al. (2020). They identified distinct types of diabetes arising from COVID-19 infection, deepening our understanding of the complexities of these intertwined conditions [3].

In terms of long-term outcomes, a study by [20] found that nearly one-third of COVID-19 patients experienced long-term symptoms such as fatigue and shortness of breath, even after hospitalisation. This highlights the importance of understanding the long-term outcomes of COVID-19 patients beyond just survival time. A study by [20] found that COVID-19 patients with severe illness who received convalescent plasma therapy had a lower risk of mortality compared to those who did not receive the therapy. This suggests that convalescent plasma therapy may be an effective intervention for improving patient outcomes.

Bambra et al. (2020) delve into gender-based health disparities brought about by the COVID-19 pandemic, drawing insights from the concept of the ‘gender health paradox’. This paradox highlights the consistent finding that men generally have higher mortality rates and shorter life expectancies, while women report higher levels of morbidity. Their study introduces the ‘gender health paradox’ and the various explanations encompassing biological, social, economic, and political factors contributing to it. They also discuss international data on gender-based inequalities in COVID-19 morbidity and mortality rates, indicating that women tend to be diagnosed more frequently, yet men exhibit higher mortality rates. They further examine the potential long-term consequences of the pandemic’s aftermath on gender-based health inequalities, focusing on the repercussions of government policy responses and the emerging economic crisis. It suggests that these factors might lead to increased mortality among men and heightened morbidity among women [21].

In another study, Muñoz-Price et al. (2020) looked into the association between race and COVID-19 outcomes, considering variables such as age, sex, socioeconomic status, and comorbidities. Their study involves 2595 adults tested for COVID-19 and explores factors related to COVID-19 positivity, hospitalisation, intensive care unit admission, mechanical ventilation, and death. The findings indicate that the Black race is associated with an increased likelihood of COVID-19 positivity (odds ratio [OR], 5.37), and this association remains after adjusting for factors like age and sex [22].

Campbel et al. (2021) [23] develop predictive models that stratify hospitalised COVID-19 patients by their risk of severe outcomes, such as ICU admission, acute respiratory distress syndrome development, or intubation. The models were designed using hierarchical ensemble classification techniques and trained on a dataset of 229 COVID-19 patients. These models utilised easily accessible information, including patient characteristics, vital signs at admission, and basic lab results. The assessment of the models was based on precision (positive predictive value) and recall (sensitivity) to determine their ability to categorise patients into increasing risk groups. The study used a separate cohort of 330 patients for validation, maintaining pre-defined test cut-offs. The results showed that the models achieved high precision, particularly in the lowest risk groups, and the proportion of severe outcomes consistently increased with escalating risk groups. Notably, attributes such as C-reactive protein, lactate dehydrogenase, and D-dimer were frequently identified as significant contributors to the risk assessments. The study concluded that machine learning-based models utilising routinely collected admission data can effectively assess the risk of severe outcomes in COVID-19 patients during hospitalisation [23].

While a substantial body of research has illuminated the retrospective post-hospitalisation COVID-19 mortality risk assessment among patients in South Africa, a notable gap exists in the comprehensive exploration of survival models, specifically tailored to this population. While studies by [24,25], among others, have delved into patient-specific characteristics and the potential of machine learning, there is still a distinct lack of studies focusing on implementing survival analysis techniques. Survival models, encompassing Kaplan-Meier estimators and Cox proportional hazards models, offer a nuanced perspective by accounting for time-to-event outcomes. These models could provide insights into the temporal patterns of mortality and uncover factors that may be time-dependent in influencing post-hospitalisation COVID-19 mortality. Furthermore, survival analysis can be particularly valuable in understanding the long-term implications of COVID-19 and capturing the dynamics of recovery and mortality beyond the immediate hospitalisation period. Addressing this gap by incorporating survival models into the assessment framework can contribute to a more comprehensive understanding of mortality risk factors and guide the development of targeted interventions.

## 3. Materials and Methods

### 3.1. Data Collection

The study area for this research is the northern part of the South Africa region, known as Limpopo province. The province has approximately 5.8 million people with diverse ethnic groups and socio-economic backgrounds. The study uses deidentified secondary data collected from electronic medical records of COVID-19 patients who were hospitalised across the health facilities in three districts, namely, Mopani, Waterberg, and Capricorn, compiled by the Department of Health in the Limpopo province between 27 June 2020 and 29 August 2022. The demographic and clinical characteristics of COVID-19 patients, including age, gender, comorbidities, and laboratory markers, were collected and treated as potential risk factors for mortality rate in the model building process. The study design is a retrospective cohort study of patients with COVID-19 infection who were followed up through 29 August 2022.

Participants for the study were identified through retrospective analysis of electronic medical records of COVID-19 patients who were hospitalised in health facilities across the Mopani, Waterberg, and Capricorn districts of the Limpopo province, South Africa. These records covered the period between 27 June 2020 and 29 August 2022. As the study focused on post-hospitalisation COVID-19 mortality risk assessment, informed consent was not required from individual participants, as data were anonymised and deidentified to ensure patient confidentiality and ethical compliance. The utilisation of existing medical records facilitated a comprehensive assessment of patients’ characteristics and outcomes within the defined timeframe, enabling a detailed exploration of mortality risk factors among the target population.

### 3.2. Summary of the Data

This study will utilise the records of patients who tested positive for COVID-19 and were hospitalised between 27 June 2020 and 29 August 2022. The sample size comprises 388 patients meeting the research criteria after data cleaning and editing. All age groups of COVID-19-positive patients hospitalised within the specified timeframe were included in the study, while patients with incomplete records were excluded. This research is conducted in accordance with ethical principles, including obtaining informed consent, ensuring confidentiality, and protecting data. Ethical clearance has been granted by the Turfloop Research Ethics Committee (TREC) and the Department of Health in Limpopo for using data in this study. While investigating mortality rates and associated risk factors among hospitalised COVID-19 patients, potential methodological errors such as measurement bias, sampling bias, confounding variables, reverse causation, and missing data were carefully assessed and addressed. To mitigate these issues, the study employed stringent patient selection, standardised data collection, confounding control, and transparent reporting practices. These efforts enhance the study’s credibility, aiming to provide reliable insights for improved post-hospitalisation COVID-19 mortality risk assessment and patient outcomes in South Africa.

### 3.3. Models

In this study, we will apply survival models to analyse the data. Survival analysis is a statistical method for analysing time-to-event data, such as the time until death and disease relapse. It is often used in medical and social sciences research to understand the factors that influence the occurrence of an event [26].

#### 3.3.1. Survival Function

The survival function describes the probability that a subject will survive beyond a certain time point. It is denoted by S(t) and is defined as the probability that the survival time T is greater than or equal to t:

(1) $$S\left(t\right)=P\left(T\geq t\right).$$

The survival function can also be expressed in terms of the cumulative distribution function (CDF) as:

(2) $$S\left(t\right)=1-F\left(t\right)$$

where F(t) is the cumulative distribution function (CDF), which gives the probability that T is less than or equal to t.

The survival function has the following properties:

▪ It is a non-increasing function of time, i.e., S(t) decreases as t increases;
▪ S(0)=1, since everyone survives at time 0;
▪ S(∞)=0, since eventually, everyone will experience the event of interest [26].

#### 3.3.2. Hazard Function

The hazard function is used in survival analysis to describe the instantaneous rate at which events occur. It is denoted by h(t) and is defined as the probability that the event of interest occurs at time t, given that it has not yet occurred up to time t, divided by the length of the time interval:

(3) h(t)=limΔt→0[P(t≤T<t+Δt|T≥t)/Δt],

where T is the survival time of interest.

The hazard function has the following properties:

▪ It is a non-negative function of time, i.e., h(t) ≥ 0 for all t;
▪ It can take on different shapes, depending on the distribution of the survival time;
▪ The area under the hazard function curve gives the total number of events expected to occur in the population during the study period.

The hazard function can be used to estimate the survival function using the relationship:

(4) $$S\left(t\right)=\exp\left(-\int_{0}^{t}h\left(u\right)du\right),$$

where the integral is taken from 0 to t. The hazard function is a useful tool in survival analysis because it allows us to model the instantaneous risk of an event, which can vary over time [26].

#### 3.3.3. Relationship between Hazard Function and Survival Function

The hazard and survival functions are two closely related functions used in survival analysis to model the time-to-event data. The survival function S(t) describes the probability that an individual survives beyond time t, while the hazard function h(t) describes the instantaneous rate at which events occur at time t.

The relationship between the two functions can be expressed mathematically as follows:

(5) $$h\left(t\right)=-\frac{d}{dt}\left[log\left(S\left(t\right)\right)\right],$$

where d/dt denotes the derivative with respect to time. This means that the hazard function is the negative derivative of the natural logarithm of the survival function. Conversely, the survival function can be obtained from the hazard function using the relationship:

(6) $$S\left(t\right)=\exp\left(-\int_{0}^{t}h\left(u\right)du\right),$$

where $\int_{0}^{t}h\left(u\right)du$ is the cumulative hazard function, which is the integral of the hazard function from 0 to t. In summary, the hazard function and survival function are complementary functions in survival analysis. The hazard function describes the instantaneous risk of an event, while the survival function describes the probability of surviving beyond a certain time.

#### 3.3.4. Estimation of Survival Function and Regression Models

##### Kaplan-Meier Model

The Kaplan-Meier method is a non-parametric method used to estimate the survival function in survival analysis. It involves calculating the probability of surviving without experiencing the event of interest at each time point using the formula:

(7) $$S\left(t\right)=\prod_{i:t_{i}\leq t}\left[1-\frac{d_{i}}{n_{i}}\right],\;$$

where S(t) is the survival function at time t, $d_{i}\;$is the number of events (e.g., deaths) that occurred before time t, and $n_{i}$ is the number of individuals still at risk of experiencing the event at time t. The Kaplan-Meier estimator produces a stepwise survival curve that is commonly used in the analysis of survival data [26].

##### Cox Proportional Hazard Model

Regression models in survival analysis are used to investigate the relationship between predictor variables and the hazard function, which represents the instantaneous risk of an event occurring over time. Various regression models are used in survival analysis, but the most common is the Cox proportional hazards model.

The Cox model assumes that the hazard function can be expressed as a product of a baseline hazard function and a function of the predictor variables, where the latter is proportional to the baseline hazard. Mathematically, the Cox proportional hazards model can be written as follows:

(8) $$h\left(t|X\right)=h_{0}\left(t\right)exp\left(X\beta \right),$$

where h(t|X) is the hazard function at time t for an individual with predictor values $X,\;h_{0}(t)$ is the baseline hazard function, β is a vector of regression coefficients and exp(.) is the exponential function. The Cox model does not require assumptions about the shape of the baseline hazard function, but it assumes that the effect of the predictor variables on the hazard is constant over time (i.e., proportional hazard assumption).

Estimation of the regression coefficients in the Cox model is typically done using partial likelihood, which accounts for censoring (i.e., when the event of interest has not yet occurred for some individuals). The partial likelihood function for the Cox model is

(9) $$L\left(\beta \right)=\prod_{i\in D}\left[\frac{exp\left(Xi\beta \right)}{\sum j}\in R_{i}\;\exp\left(X_{j}\;\beta \right)\right],$$

where D is the set of individuals who experience the event of interest, $R_{i}$ is the set of individuals who are at risk of experiencing the event at the time of observation for individual i. The partial likelihood estimate of β is obtained by maximising the partial likelihood function [27,28,29].

##### Frailty Models

Frailty models are used in survival analysis to account for unobserved heterogeneity or clustering of individuals within groups, especially when the proportional hazard assumption is violated. The basic idea of frailty models is to introduce a random effect term that captures the unobserved heterogeneity among individuals within a group. The random effect is assumed to follow a specific distribution, such as a normal or gamma distribution, and is incorporated into the hazard function.

Mathematically, the frailty model can be written as:

(10) $$h\left(t|Z,\vartheta \right)=h_{0}\left(t\right)exp\left(Z\beta +\gamma u\right),$$

where h(t|Z,ϑ) is the hazard function at time t for an individual with covariate values Z, β is a vector of regression coefficients, u is the frailty term that captures the unobserved heterogeneity, and γ is the scaling parameter that determines the strength of the effect of the frailty term. The frailty term u is assumed to follow a specific distribution, such as a normal or gamma distribution, with mean 1 and variance ϑ.

The likelihood function for the frailty model is a mixed-effects model, which involves integrating over the distribution of the frailty term. The likelihood function can be written as:

(11) $$L(\beta ,\vartheta )=\prod_{i=1}^{n}\left[h{\left(t_{i}|Z_{i},\vartheta \right)}^{\left(\delta_{i}\right)}S{\left(t_{i}|Z_{i},\vartheta \right)}^{\left(1-\delta_{i}\right)}f\left(u|\vartheta \right)\right],\;$$

where n is the number of individuals,$\;t_{i}$ is the survival time for individual i, $Z_{i}$ is the covariate vector for individual i, $\delta_{i}$ is the censoring indicator ($\delta_{i}=1$ if the event is observed and $\delta_{i}=0$ if the event is censored), $S(t_{i}|Z_{i},\vartheta )$ is the survival function for individual i, and f(u|ϑ) is the density function of the frailty term u. Estimation of the parameters in the frailty model can be done using maximum likelihood estimation or Bayesian methods [22,23].

##### Parametric Models

Parametric survival models are used to model the time to an event of interest, such as death or disease recurrence, using a set of covariates. The baseline hazard function represents the probability of the event occurring without any covariates, and the covariate effects are captured by the regression coefficients. In this methodology, we will focus on three common parametric models with different baseline hazard functions: the generalised gamma, gamma, and Weibull models.

The generalised gamma model allows for flexible shapes of the baseline hazard function, making it suitable for modelling survival data with various patterns of hazard rates. The baseline hazard function of the generalised gamma model is defined as:

(12) $$h\left(t\right)=\left(\frac{a}{b}\right){\left(\frac{t}{c}\right)}^{\left(a-1\right)}*\;exp{\left({-\left(\frac{t}{c}\right)}^{b}\right)}^{a},$$

where a, b, and c are shape parameters. The regression coefficients are estimated using maximum likelihood estimation.

The gamma model is a special case of the generalised gamma model with b=1, which results in a simpler baseline hazard function:

(13) $$h\left(t\right)=\left(\frac{a}{b}\right){\left(\frac{t}{c}\right)}^{\left(a-1\right)}*\exp\left[-(t/c\right)],$$

where a and c are shape parameters. The gamma model is particularly useful for modelling survival data with a monotonically decreasing hazard rate.

The Weibull model has a baseline hazard function that is an exponential function of time, which makes it suitable for modelling survival data with an increasing hazard rate. The baseline hazard function of the Weibull model is defined as:

(14) $$h\left(t\right)=a\;b\;*\;t^{\left(b-1\right)},$$

where a and b are shape parameters. The regression coefficients are estimated using maximum likelihood estimation. In conclusion, parametric survival models with generalised gamma, gamma, and Weibull baseline hazards are useful for modelling survival data with different patterns of hazard rates [27,28,29].

### 3.4. Analysis

The statistical analysis was performed using R version 4.0.3 and SPSS IBM Statistics version. For continuous variables following a parametric distribution, they were presented as mean ± standard deviation (SD), while nonparametric variables were expressed as medians ± interquartile range. Categorical variables were presented as counts and percentages, and crosstabulation. To analyse the data, various semiparametric survival models, including Cox proportional regression, frailty models, and parametric models, were applied, and their fit was compared using the loglikelihood. A significance level of *p* < 0.05 was used to determine statistical significance.

## 4. Results and Discussion

This section presents the study results and discussion, utilising secondary data from the Department of Health in the Limpopo province in South Africa. The data were collected on patients who had COVID-19 and symptoms of the disease admitted across Limpopo province hospitals, and the data is accessed in Microsoft Excel format. Table 1 presents the description of the variables and the dataset codes. Data for survival regression models require a specific structure, and variables that did not meet the standard were omitted from the analysis.

At the outset of our analysis, we initiated an extensive exploration of the dataset utilizing descriptive statistics, as demonstrated in Table 2. This fundamental phase provides us with a valuable opportunity to grasp the distribution and grouping of patients across various categories. Through our engagement with descriptive statistics, we establish a solid groundwork for our ensuing analyses, facilitating the discovery of inherent patterns, emerging trends, and prospective insights that will underpin our research endeavours.

**Figure 1:**
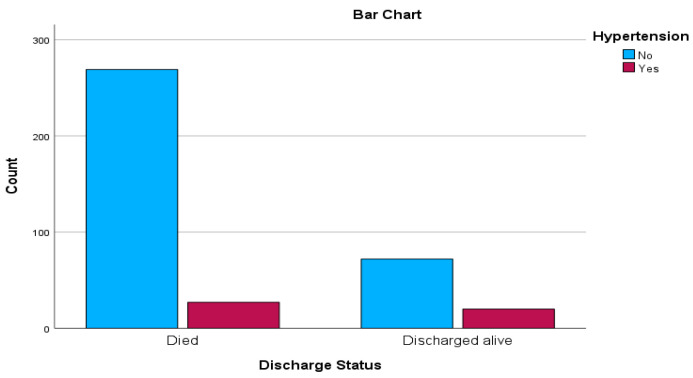
Bar chart depicting the distribution of hypertension and discharged status.

The descriptive statistics from Table 2 and Figure 1 offer valuable insights into the characteristics of the study population and their relevance to the topic in terms of mortality rates and risk factors associated with COVID-19. The descriptive statistics tables provide a comprehensive overview of various aspects within the context of the research on retrospective post-hospitalisation COVID-19 mortality risk assessment of patients in South Africa. These tables offer insights into different variables related to the study population. In terms of gender distribution, 52.6% of patients were female, and 47.4% were male. The distribution across facility types indicates that 49.2% were in private general hospitals, 42.5% in district hospitals, and 8.2% in provincial tertiary hospitals. Regarding districts, 68.8% of patients were from Capricorn, 27.8% from Mopani, and 3.4% from Waterberg. The outcomes of hospitalisation show that 76.3% of patients died in the hospital, while 23.7% were discharged alive. Comorbidity prevalence is detailed across Table 2, with conditions like hypertension (87.9% without, 12.1% with), diabetes (83.5% without, 16.5% with), asthma (89.9% without, 10.1% with), chronic pulmonary disease (94.1% without, 5.9% with), tuberculosis (90.7% without, 9.3% with) (Table 2), and obesity (86.3% without, 13.7% with). Oxygenation (46.4% received oxygen, 53.6% did not) and ventilation (18.6% received ventilation, 81.4% did not) during hospitalisation are also highlighted. These statistics collectively provide a comprehensive understanding of the demographic, clinical, and intervention-related characteristics of the study population, contributing to a comprehensive assessment of COVID-19 mortality risk among patients in South Africa.

**Figure 2:**
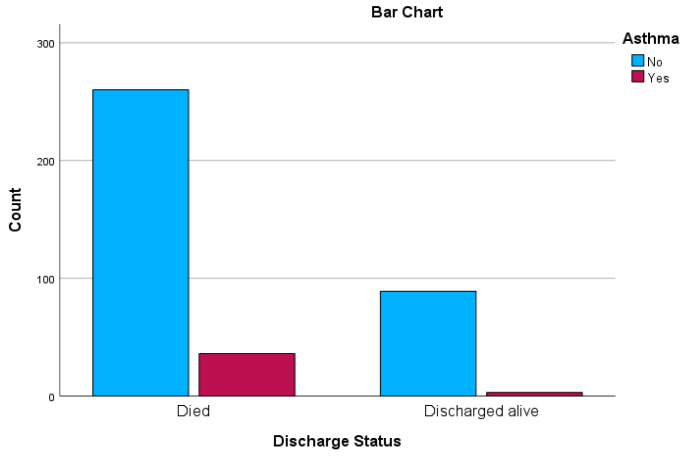
Bar chart depicting the distribution of asthma and discharged status.

In the upcoming sections, we introduce crosstabulation as an integral component of our results and discussion presentation, with the intention of delving further into the intricate interplay between variables. This analytical methodology provides us with the means to investigate the mutual influence of diverse factors on outcomes, thereby revealing potential associations that may be obscured otherwise. Through the utilisation of crosstabulation, we have the opportunity to unearth subtle insights that enrich our comprehension of the intricate dynamics inherent to our research domain. These results are presented in Table 3 and Table 4, together with graphs in Figure 1 and Figure 2 displaying the distribution of hypertension and asthma in relation to discharged status.

Table 3 presents the results of Chi-Square tests assessing the association between the presence of hypertension and the discharge status of patients in the context of the research. These tests aim to determine whether there is a significant relationship between the two variables—hypertension and discharged status—and to ascertain if the presence of hypertension has an impact on the likelihood of being discharged alive or dying after hospitalisation.

The Pearson Chi-Square test statistic is 10.496 with one degree of freedom, yielding an asymptotic significance level of 0.001. This indicates a statistically significant relationship between hypertension and discharged status. The Continuity Correction Chi-Square value is 9.344 with the same degrees of freedom and a significance level of 0.002, which further strengthens the evidence of a significant association. The Likelihood Ratio test also yields a Chi-Square value of 9.380 with one degree of freedom and a significance level of 0.002.

The Fisher’s Exact Test, often employed when dealing with small cell sizes, demonstrates a significant exact two-sided *p*-value of 0.003 and a significant one-sided *p*-value of 0.002. This indicates a notable relationship between hypertension and discharged status, even when considering smaller sample sizes or rare occurrences.

Similarly, Table 4 presents the outcomes of Chi-Square tests investigating the association between the presence of asthma and the discharge status of patients within the research context. These tests aim to establish whether there is a significant relationship between these two variables and whether the presence of asthma affects the likelihood of being discharged alive or dying following hospitalisation.

The Pearson Chi-Square test statistic results in a value of 6.151 with one degree of freedom, yielding a two-sided asymptotic significance level of 0.013. This indicates a statistically significant connection between asthma and discharged status, suggesting that asthma might influence patient outcomes after hospitalisation for COVID-19. Similarly, the Continuity Correction Chi-Square value is 5.206 with a significance level of 0.023, strengthening the evidence of a significant association.

The Likelihood Ratio test yields a Chi-Square value of 7.577 with one degree of freedom and a significance level of 0.006. This further emphasises the statistically significant relationship between asthma and discharge status.

The Fisher’s Exact Test, designed for small cell sizes, exhibits an exact two-sided *p*-value of 0.010 and a one-sided *p*-value of 0.007. These small *p*-values suggest a noteworthy association between asthma and the likelihood of being discharged or dying after COVID-19 hospitalisation, even when considering smaller sample sizes or rare occurrences.

Importantly, all expected cell counts in the table are greater than 5, and the minimum expected count is 9.25, satisfying the prerequisite for the application of Chi-Square tests. The tests are computed for a 2 × 2 table.

In summary, the findings of Table 4 underscore a statistically significant link between the presence of asthma and the discharge status of patients post-hospitalisation. This suggests that asthma might have a discernible impact on the outcomes of COVID-19 patients, potentially influencing the course of their recovery or mortality. These results hold implications for healthcare strategies and patient care approaches, warranting further investigation into the relationship between asthma and COVID-19 outcomes.

Before delving into the application of semiparametric and parametric models, we begin our analysis by examining the Kaplan-Meier survival function. Initially, we explore the overall survival function to gain a holistic view of the mortality trends within our dataset. Additionally, we dissect this function based on gender, allowing us to discern potential disparities in survival probabilities between male and female patients. These preliminary steps pave the way for a comprehensive investigation of our data, enabling us to subsequently fit semiparametric and parametric models. We aim to unravel the complex interplay of variables influencing COVID-19 mortality outcomes through these models.

**Figure 3:**
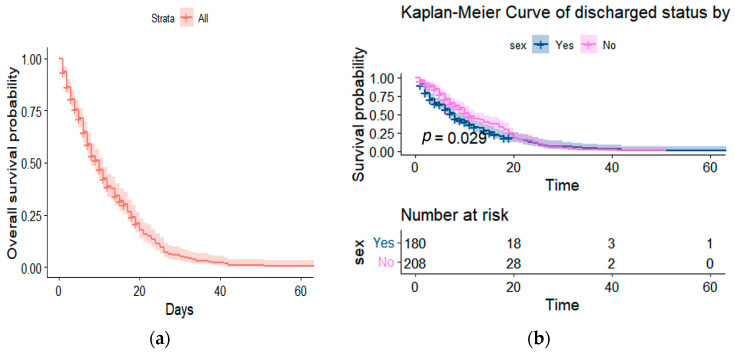
Survival functions for (**a**) all the patients and (**b**) gender using the Kaplan-Meier method.

It is also evident from Figure 3 that the overall survival of patients for the first 60 days declines steadily, with the survival function amongst males and females being significant, with a *p*-value of 0.029.

In our pursuit to comprehensively understand the intricate landscape of COVID-19 mortality risk assessment among post-hospitalised patients in South Africa, we turn our attention to the fitting of semiparametric and parametric models. These advanced analytical techniques hold the potential to unravel the multifaceted associations between various risk factors and mortality outcomes. By applying semiparametric and parametric models, we endeavour to go beyond descriptive insights, delving into the underlying mechanisms that drive mortality trends. Through this approach, we aspire to unearth valuable insights that can inform targeted interventions and strategies for improving patient outcomes within the context of the South African healthcare landscape. These models were compared using the loglikelihood after checking the underlying assumptions. The selected model is presented in Table 5, whereas the competitive models are presented in Appendix A.

When comparing the models and selecting the best-fitted one based on loglikelihood and AIC, it is essential to consider the assumptions and clinical relevance of the models in addition to their statistical fit. The frailty regression model presented in Table 5 has a higher loglikelihood −1237.52 compared to the parametric survival models displayed in Appendix A. Again, the frailty regression model has the advantage of directly accounting for frailty, which may have clinical relevance to our problem statement. This model assumes a random effect for frailty, allowing for individual heterogeneity in the baseline hazard.

Table 5 presents a comprehensive analysis of parameter estimates within the frailty regression model, offering valuable insights into the mortality risk assessment of post-hospitalised COVID-19 patients in the context of the research topic. Each parameter estimate, along with its associated Hazard Ratio (HR), 95% Confidence Interval (CI), and *p*-value, provides a detailed understanding of the impact of various characteristics on mortality outcomes.

Among the factors examined, the presence of asthma does not appear to exert a statistically significant influence on mortality risk, as evidenced by the non-significant HR of 0.83 (95% CI: 0.48 to 1.46, *p* = 0.5). This suggests that asthma might not play a substantial role in determining COVID-19-related mortality among the studied patients. In contrast, diabetes emerges as a significant contributor to mortality risk, with patients having a 1.69 times higher hazard of mortality (95% CI: 1.03 to 2.76, *p* = 0.038) compared to those without diabetes. This emphasises the importance of managing diabetes as a crucial comorbidity to mitigate the risk of adverse outcomes following COVID-19 hospitalisation.

Hypertension stands out as a notable risk factor, significantly elevating the hazard of mortality. Patients with hypertension exhibit a substantial 3.31 times higher hazard of mortality (95% CI: 1.61 to 6.81, *p* = 0.001), underscoring the critical need to address this comorbidity in COVID-19 patient management strategies. Intriguingly, tuberculosis seems to have a protective effect, resulting in a reduced hazard of mortality among affected patients. The HR of 0.38 (95% CI: 0.18 to 0.79, *p* = 0.010) implies that individuals with tuberculosis experience a lower risk of mortality, suggesting potential immune responses or effective medical interventions associated with tuberculosis that warrant further investigation.

Notably, obesity does not significantly alter mortality risk among the studied patients, as indicated by an HR of 0.74 (95% CI: 0.42 to 1.30, *p* = 0.3). This implies that obesity might not play a prominent role in influencing mortality outcomes in this specific context. Geographic disparities are also evident, with patients from the Capricorn district facing an alarmingly higher hazard of mortality (HR: 17.1, 95% CI: 7.61 to 38.3, *p* < 0.001) compared to the reference district, Mopani. This highlights the need to address regional variations in healthcare access and outcomes.

Medical interventions play a significant role as well. Ventilation during hospitalisation is associated with a substantial reduction in the hazard of mortality (HR: 0.34, 95% CI: 0.22 to 0.54, *p* < 0.001), underlining its critical importance in the treatment of severe COVID-19 cases. Gender differences also come to the forefront, with males having a higher hazard of mortality (HR: 1.55, 95% CI: 1.09 to 2.21, *p* = 0.015), suggesting potential gender-related disparities in COVID-19 outcomes.

In the broader context of our problem, these parameter estimates provide invaluable insights into the intricate interplay between various characteristics and mortality risks among post-hospitalised COVID-19 patients. These findings hold profound implications for healthcare interventions and patient management strategies. Addressing diabetes, hypertension, regional disparities, and medical interventions such as ventilation become imperative for improving outcomes. Overall, these parameter estimates significantly contribute to our understanding of COVID-19 mortality risk assessment and offer crucial guidance for tailored strategies to enhance patient care and outcomes in South Africa.

In relation to previous research, diabetes is identified as a significant risk factor for mortality, aligning with previous studies that have consistently reported the detrimental impact of diabetes on COVID-19 outcomes [20,30,31]. Similarly, hypertension is found to be strongly associated with increased mortality risk, corroborating evidence suggesting hypertension as a risk factor for severe illness in COVID-19 patients [30,31]. Nonetheless, these results have implications for risk stratification and targeted interventions to improve patient care and outcomes. They can inform healthcare providers in identifying and providing appropriate care to individuals with diabetes and hypertension who are at higher risk of mortality. Additionally, the findings highlight the need for integrated care for individuals with comorbidities to optimise COVID-19 management and reduce mortality rates. Further studies are warranted to validate these findings and explore potential mechanisms underlying the observed associations.

## 5. Conclusions and Implication of Results

In conclusion, the findings of this study offer comprehensive insights into the intricate web of factors shaping post-hospitalisation COVID-19 mortality risk among patients in South Africa’s Limpopo province. The robust association between diabetes and hypertension with increased mortality rates underscores the imperative of effectively managing these underlying conditions to enhance patient outcomes. The intriguing protective effect linked to tuberculosis warrants further exploration, potentially unlocking novel avenues for understanding immune responses and potential therapeutic interventions. The regional disparities, evident through elevated mortality risk in the Capricorn district, highlight the urgency of targeted interventions to address healthcare inequities across different geographical areas.

This study brings to the forefront the pivotal role of medical interventions, particularly ventilation, in significantly mitigating mortality risk. Furthermore, gender-based variations in mortality outcomes underscore the necessity of tailored approaches to ensure gender-specific healthcare strategies. These findings carry significant implications for healthcare interventions and patient management strategies. Prioritizing the management of diabetes, hypertension, and regional disparities could lead to substantial improvements in patient outcomes. The protective effect of tuberculosis holds promise for innovative therapeutic avenues, while emphasising the importance of understanding diverse immune responses.

Overall, this study provides actionable insights that can inform healthcare policies, allocation of resources, and clinical practices to effectively address and mitigate COVID-19 mortality risk among post-hospitalised patients in South Africa’s Limpopo province. The results underscore the importance of a holistic and tailored approach to patient care, offering a valuable roadmap for both current and future efforts in tackling the challenges posed by the pandemic.

## Figures and Tables

**Table 1:** Variable description and dataset codes.

Variable	Description
Discharged status	Binary response from the patients: discharged alive, died.
District	The three districts: Mopani, Capricorn, and Waterberg
Gender/Sex	Gender of a participant: Female and Male
Chronic diseases	Hypertension, Diabetes, Asthma, Chronic Pulmonary Disease, Tuberculosis, Obesity (with two levels each, yes, no)
Ever Oxygenated	yes or no
Ever Ventilated	yes or no
Obesity	yes, no, unknown
Facility Type	Private General Hospital District Hospital, Provincial Tertiary Hospital

NB: This table displays all the variables used in the modelling process and their codes.

**Table 2:** Descriptive statistics.

Category	Frequency	Percent	Valid Percent	Cumulative Percent
**Sex/Gender**				
F	204	52.6	52.6	52.6
M	184	47.4	47.4	100.0
Total	388	100.0	100.0	
**Facility Type**				
District Hospital	165	42.5	42.5	42.5
Private General Hospital	191	49.2	49.2	91.8
Provincial Tertiary Hospital	32	8.2	8.2	100.0
Total	388	100.0	100.0	
**Districts**				
Capricorn	267	68.8	68.8	68.8
Mopani	108	27.8	27.8	96.6
Waterberg	13	3.4	3.4	100.0
Total	388	100.0	100.0	
**Discharge Status**				
Died	296	76.3	76.3	76.3
Discharged alive	92	23.7	23.7	100.0
Total	388	100.0	100.0	
**Hypertension**				
No	341	87.9	87.9	87.9
Yes	47	12.1	12.1	100.0
Total	388	100.0	100.0	
**Diabetes**				
No	324	83.5	83.5	83.5
Yes	64	16.5	16.5	100.0
Total	388	100.0	100.0	
**Asthma**				
No	349	89.9	89.9	89.9
Yes	39	10.1	10.1	100.0
Total	388	100.0	100.0	
**Chronic Pulmonary Disease**				
No	365	94.1	94.1	94.1
Yes	23	5.9	5.9	100.0
Total	388	100.0	100.0	
**Tuberculosis**				
No	352	90.7	90.7	90.7
Yes	36	9.3	9.3	100.0
Total	388	100.0	100.0	
**Obesity**				
No	335	86.3	86.3	86.3
Yes	53	13.7	13.7	100.0
Total	388	100.0	100.0	
**Ever Oxygenated**				
No	208	53.6	53.6	53.6
Yes	180	46.4	46.4	100.0
Total	388	100.0	100.0	
**Ever Ventilated**				
No	316	81.4	81.4	81.4
Yes	72	18.6	18.6	100.0
Total	388	100.0	100.0	

**NB**: The descriptive statistics depicting frequencies of all variables.

**Table 3:** Crosstabulation between hypertension and sischarged status.

	Value	df	Asymptotic Significance (2-Sided)	Exact Sig. (2-Sided)	Exact Sig. (1-Sided)
Pearson Chi-Square	10.496 ^a^	1	0.001		
Continuity Correction ^b^	9.344	1	0.002		
Likelihood Ratio	9.380	1	0.002		
Fisher’s Exact Test				0.003	0.002
N of Valid Cases	388				

^a^. 0 cells (0.0%) have expected count less than 5. The minimum expected count is 11.14. ^b^. Computed only for a 2 × 2 table. H_0_: There is no relationship between the two variables.

**Table 4:** Crosstabulation between asthma and discharged status.

	Value	df	Asymptotic Significance (2-Sided)	Exact Sig. (2-Sided)	Exact Sig. (1-Sided)
Pearson Chi-Square	6.151 ^a^	1	0.013		
Continuity Correction ^b^	5.206	1	0.023		
Likelihood Ratio	7.577	1	0.006		
Fisher’s Exact Test				0.010	0.007
N of Valid Cases	388				

^a^. 0 cells (0.0%) have expected count less than 5. The minimum expected count is 9.25. ^b^. Computed only for a 2 × 2 table. H_0_: There is no relationship between the two variables.

**Table 5:** Parameter estimates for the frailty regression model.

Characteristic	HR ^1^	95% CI ^1^	*p*-Value
Asthma			
No	—	—	
Yes	0.83	0.48, 1.46	0.5
Diabetes			
No	—	—	
Yes	1.69	1.03, 2.76	**0.038**
Hypertension			
No	—	—	
Yes	3.31	1.61, 6.81	**0.001**
Tuberculosis			
No	—	—	
Yes	0.38	0.18, 0.79	**0.010**
Obesity			
No	—	—	
Yes	0.74	0.42, 1.30	0.3
District			
Mopani	—	—	
Capricorn	17.1	7.61, 38.3	**<0.001**
Waterberg	0.41	0.05, 3.50	0.4
Ever Oxygenated			
Yes	—	—	
No	0.95	0.60, 1.50	0.8
Ever Ventilated			
No	—	—	
Yes	0.34	0.22, 0.54	**<0.001**
Sex			
F	—	—	
M	1.55	1.09, 2.21	**0.015**
*Frailty (Column1)*			*<0.001*
^1^ HR = Hazard Ratio, CI = Confidence Interval, Global *p*-value = 1.00, Loglikelihood = −1237.52, AIC = 2743.64			

**NB**: The Global *p*-value evaluates the hypothesis underlying the model, where H_0_: there is no association between the term and the response.

**Figure A1:**
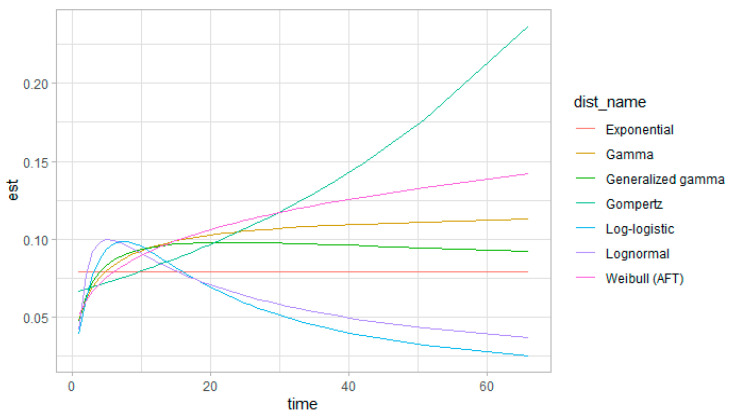
Parametric distribution fits for discharged status.

## Data Availability

The data can be shared upon request from the authors and the Department of Health.

## Appendix A

**Table A1:** Parameter estimates for Cox proportional hazard regression model.

Characteristic	HR ^1^	95% CI ^1^	*p*-Value
Asthma			
No	—	—	
Yes	0.99	0.67, 1.46	>0.9
Diabetes			
No	—	—	
Yes	1.17	0.83, 1.65	0.4
Hypertension			
No	—	—	
Yes	2.49	1.43, 4.36	**0.001**
Tuberculosis			
No	—	—	
Yes	0.54	0.31, 0.93	**0.027**
Obesity			
No	—	—	
Yes	0.98	0.66, 1.45	>0.9
Chronic Pulmonary Disease			
No	—	—	
Yes	1.08	0.56, 2.09	0.8
Facility Type			
District Hospital	—	—	
Private General Hospital	0.46	0.30, 0.69	**<0.001**
Provincial Tertiary Hospital	0.40	0.25, 0.66	**<0.001**
District			
Mopani	—	—	
Capricorn	7.82	4.26, 14.3	**<0.001**
Waterberg	0.38	0.05, 2.86	0.3
Ever Oxygenated			
Yes	—	—	
No	0.88	0.64, 1.21	0.4
Ever Ventilated			
No	—	—	
Yes	0.60	0.45, 0.81	**<0.001**
Sex			
F	—	—	
M	1.15	0.91, 1.46	0.2
^1^ HR = Hazard Ratio, CI = Confidence Interval, Global *p*-value = 0.00, Loglikelihood = −1409, AIC = 2844.00			

**Table A2:** Parameter estimates for parametric survival model with Gamma baseline hazard distribution.

Characteristic	HR ^1^	Beta	95% CI ^1^	*p*-Value
shape	--	1.55	1.34, 1.79	--
rate	--	0.06	0.04, 0.10	--
Asthma				
Yes	0.99	−0.01	−0.33, 0.30	0.5
No	--			
Diabetes				
Yes	1.13	0.12	−0.14, 0.39	0.2
No	--			
Hypertension				
Yes	1.97	0.68	0.25, 1.11	**<0.001**
No	--			
Tuberculosis				
Yes	0.60	−0.51	−0.93, −0.08	**0.009**
No	--			
Obesity				
Yes	0.98	−0.02	−0.33, 0.28	0.4
No	--			
Chronic Pulmonary Disease				
Yes	1.08	0.08	−0.43, 0.59	0.4
No				
Facility type				
Private General Hospital	0.50	−0.69	−1.02, −0.36	**<0.001**
Provincial Tertiary Hospital	0.46	−0.77	−1.15, −0.39	**<0.001**
District Hospital	--			
Ever Oxygenated				
No	0.91	−0.09	−0.34, 0.17	0.3
Yes	--			
Ever Ventilated				
Yes	0.66	−0.41	−0.64, −0.18	**<0.001**
No	--			
Sex				
M	1.12	0.12	−0.07, 0.30	0.11
F				
District				
Capricorn	4.71	1.55	1.08, 2.02	**<0.001**
Waterberg	0.52	−0.66	−2.10, 0.77	0.2
Mopani	---			
HR ^1^ = Hazard Ratio, ^1^ CI = Confidence Interval, Loglikelihood = −996.33, AIC = 2022.67				

**Table A3:** Parameter estimates for parametric survival model with Gen Gamma baseline hazard distribution.

Characteristic	HR ^1^	Beta	95% CI ^1^	*p*-Value
mu	--	2.97	2.56, 3.39	
sigma	--	0.88	0.80, 0.96	
Q	--	0.31	0.02, 0.60	
Asthma				
Yes	1.09	0.09	−0.24, 0.43	0.3
No	--			
Diabetes				
Yes	0.79	−0.23	−0.51, 0.04	**0.049**
No	--			
Hypertension				
Yes	0.53	−0.63	−1.04, −0.21	**0.001**
No	--			
Tuberculosis				
Yes	1.73	0.55	0.12, 0.97	**0.006**
No	--			
Obesity				
Yes	1.09	0.09	−0.23, 0.42	0.3
No				
Chronic Pulmonary Disease				
Yes	0.94	−0.06	−0.58, 0.45	0.4
No				
Facility Type				
Private General Hospital	2.08	0.73	0.37, 1.08	**<0.001**
Provincial Tertiary Hospital	1.84	0.61	0.18, 1.04	**0.003**
District Hospital	--			
Ever Oxygenated				
No	1.07	0.07	−0.19, 0.33	0.3
Yes	--			
Ever Ventilated				
Yes	1.69	0.53	0.27, 0.79	**<0.001**
No	--			
Sex				
M	0.83	−0.18	−0.37, 0.02	**0.040**
F				
District				
Capricorn	0.21	−1.54	−1.99, −1.09	<0.001
Waterberg	1.89	0.64	−0.57, 1.84	0.15
Mopani	--			
HR ^1^ = Hazard Ratio, ^1^ CI = Confidence Interval, Loglikelihood = −991.02, AIC = 2014.04				

**Table A4:** Parameter estimates for parametric survival model with Weibull baseline hazard distribution.

Characteristic	HR ^1^	Beta	95% CI ^1^	*p*-Value
shape	--	1.25	1.15, 1.36	
scale	--	27.9	17.8, 43.5	
Asthma				
Yes	1.00	0.00	−0.31, 0.31	0.5
No	--			
Diabetes				
Yes	0.91	−0.09	−0.36, 0.19	0.3
No	--			
Hypertension				
Yes	0.49	−0.72	−1.17, −0.27	**<0.001**
No	--			
Tuberculosis				
Yes	0.65	0.50	0.07, 0.94	**0.012**
No	--			
Obesity				
Yes	1.00	0.00	−0.32, 0.31	0.5
No				
Chronic Pulmonary Disease				
Yes	0.92	−0.08	−0.61, 0.44	0.4
No				
Facility Type				
Private General Hospital	1.92	0.65	0.32, 0.98	**<0.001**
Provincial Tertiary Hospital	2.25	0.81	0.43, 1.20	**<0.001**
District Hospital	--			
Ever Oxygenated				
No	1.05	0.10	−0.15, 0.35	0.2
Yes	--			
Ever Ventilated				
Yes	1.46	0.38	0.15, 0.61	<0.001
No	--			
Sex				
M	0.91	−0.09	−0.28, 0.10	0.2
F	--			
District				
Capricorn	0.21	−1.58	−2.08, −1.08	**<0.001**
Waterberg	2.09	0.74	−0.88, 2.35	0.2
Mopani	--			
HR ^1^ = Hazard Ratio, ^1^ CI = Confidence Interval, Loglikelihood = −1000.49, AIC = 2030.99				

**Table A5:** Criteria for selecting the best-fitted distribution for the parametric models.

Distribution	Akaike Information Criterion (AIC)
**Gamma**	**2075.2**
**Generalised Gamma**	**2077.3**
**Weibull**	**2078.4**
Gompertz	2089.5
LogNormal	2091.0
Exponential	2098.0
LogLogistic	2100.0
